# Impact of Media Coverage on Influenza Vaccine Coverage in Elderly Individuals from 2020 to 2021 in the Republic of Korea

**DOI:** 10.3390/vaccines9040367

**Published:** 2021-04-10

**Authors:** Yunhyung Kwon, Young June Choe, Jae-Won Yun, Hee Kyoung Kim, Sungnam Kim, Chaemin Chun, Yeon Haw Jung, HeeJung Kim, Hyun-kyung Oh, Yeonkyoeng Lee, Jae Young Lee, Seon Kui Lee, Young-Joon Park

**Affiliations:** 1 Director for Epidemiological Investigation Analysis, Korea Disease Control and Prevention Agency, Cheongju 28159, Korea; yhhodori@korea.kr (Y.K.); yunjw@korea.kr (J.-W.Y.); kyoung0309@korea.kr (H.K.K.); ksn1907@korea.kr (S.K.); chaem92@korea.kr (C.C.); yhjung04@korea.kr (Y.H.J.); spleen55@korea.kr (H.K.); ohhk525@korea.kr (H.-k.O.); brightlee97@korea.kr (Y.L.); pgzdtk198@korea.kr (J.Y.L.); byuly74@korea.kr (S.K.L.); 2 Department of Pediatrics, Korea University Anam Hospital, Seoul 02841, Korea; choey@korea.ac.kr

**Keywords:** influenza, vaccine, vaccination, safety, death, mortality

## Abstract

Increased awareness of adverse events following immunization (AEFI) can disrupt vaccination programs. In South Korea, a report of alleged influenza vaccine-related deaths attracted significant media attention in 2020. We retrieved the vaccination coverage and AEFI data to determine their association with media coverage. Between 2015 and 2019, the vaccination coverage rate ranged between 80.5% and 83.3%; however, the vaccination coverage rate declined significantly from 2020 to 2021 to 73.6% (*p* < 0.0001). During the 43rd week of 2020, following a large amount of media coverage on vaccine safety issues, the number of cases with AEFI reached 60. Between 2015 and 2020, the mortality rate ratios for influenza vaccines and non-vaccines ranged between 0.1296 (95% confidence interval (CI), 0.1262–0.1331, *p* < 0.0001) and 0.1608 (95% CI, 0.1572–0.1644, *p* < 0.0001). Vaccine safety surveillance should be continued in conjunction with investigation and transparent risk communication to maintain public trust in vaccines and vaccinations.

## 1. Introduction

Increased awareness of the adverse events following immunization (AEFI) may disrupt vaccination programs, which may lead to the resurgence of vaccine-preventable diseases [1]. Therefore, it is essential for public health professionals to be fully aware of any potential risk of adverse events and to actively spread safety information to ensure the success of vaccination programs. In South Korea, a report of alleged influenza vaccine-related deaths has attracted significant media attention and has affected the societal acceptance of vaccines [2]. In October 2020, the media reported a total of 28 deaths after vaccination in Korea. The deaths were not limited to any specific product nor had they any geographical clusters; however, these deaths impacted the vaccine coverage of the public immunization program. In the present study, we aimed to describe the trend of media coverage on vaccine safety alongside AEFI reporting and vaccination coverage rate and to examine the background mortality rates following influenza vaccination in the elderly population in South Korea. This retrospective study also aimed to examine the (1) ecological changes in vaccine coverage in respective seasons and (2) the differences in mortality between vaccinated and unvaccinated individuals over the past six influenza seasons.

## 2. Methods

The Korea Disease Control and Prevention Agency (KDCA) institutes vaccination policies, coordinates the operation of vaccination programs, gathers registry data to measure the coverage rate, and conducts vaccine safety investigations [3]. All data of eligible individuals and documented residents receiving influenza vaccines under the national immunization program were recorded in the computerized immunization registry, which is required for reimbursement of the vaccine and vaccination fee. Vaccination is not mandatory but is recommended by public health agencies and medical societies. The Korean influenza national immunization program was implemented to provide free influenza vaccination to adults aged ≥65 years since 2005. After handing over the influenza vaccination for elderly adults to the private sector in 2015, the influenza vaccination coverage rate among this population increased to >80% [3].

We first retrieved the data of the yearly influenza vaccination coverage rate among elderly individuals aged ≥65 years from the national influenza vaccination program from the 2015–2016 season to the 2020–2021 season. Because the study was conducted in late November 2020, the data were collected from the first week of October (the usual start of the influenza vaccination campaign for the corresponding season) to the second week of November. The numerator comes from the immunization registry, which collects the vaccination status and vaccination date of the target person. The denominator comes from the 2015 census data of all individuals aged ≥65 years who are potentially eligible for influenza vaccination. We then plotted the yearly number of AEFI cases reported to the KDCA. Then, we retrieved the weekly number of press articles in 2020 that contained the following keywords from the news big data crawler of the Korea Press Foundation: (“influenza” AND “vaccine” OR “vaccination” OR “immunization” AND “safety” OR “death” OR “mortality”). The weekly number of AEFIs reported to the KDCA was plotted in the corresponding week. Finally, we calculated the mortality rate during the first week of October and the second week of November for elderly individuals aged ≥65 years in the 2015–2016 season and the 2020–2021 season using the administrative data from the Ministry of Interior and Safety linked with the vaccination data. The age-specific mortality rate was calculated per 100,000 background population derived from the census data of the respective age groups, and the mortality rate ratio between individuals vaccinated with the influenza vaccine and unvaccinated individuals was calculated. This study was a part of the vaccine safety management activity using de-identifiable cluster data under the Infectious Disease Prevention Act and was not considered research; therefore, it was exempted from ethical review.

## 3. Results

**Figure 1:**
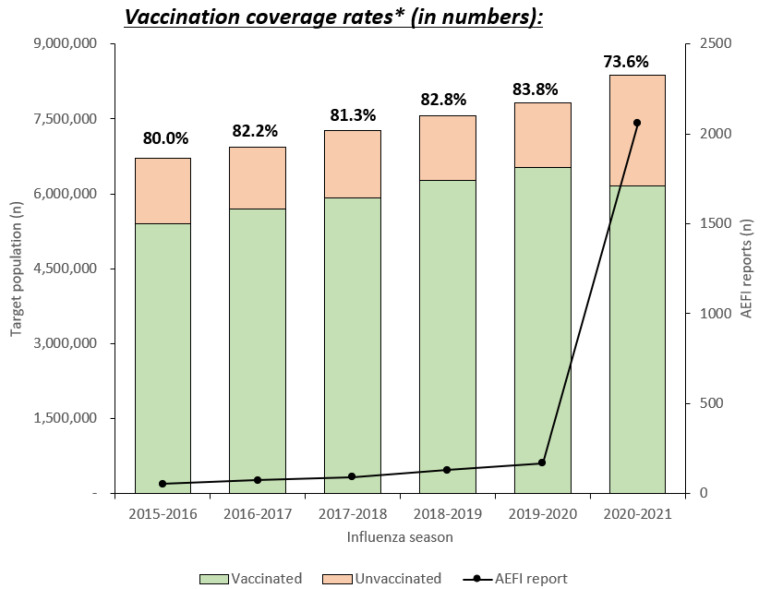
Influenza vaccination coverage among elderly individuals aged ≥65 years in South Korea in the 2015–2016 and 2020–2021 seasons. * Vaccination coverage between the first week of October and the second week of November in the corresponding seasons.

Figure 1 shows the influenza vaccination coverage among elderly individuals between 2015 and 2021. The vaccination coverage is between the first week of October and the second week of November of the corresponding seasons. The vaccination coverage ranged between 80.0% (2015–2016 season) and 83.8% (2019–2020 season); however, there was a significant decline in the vaccination coverage in the 2020–2021 season to 73.6% (*p* < 0.0001). The number of AEFI reports ranged between 54 and 170 in the 2015–2016 and 2019–2020 seasons, whereas it increased to 2059 during the corresponding weeks in the 2020–2021 season.

**Figure 2:**
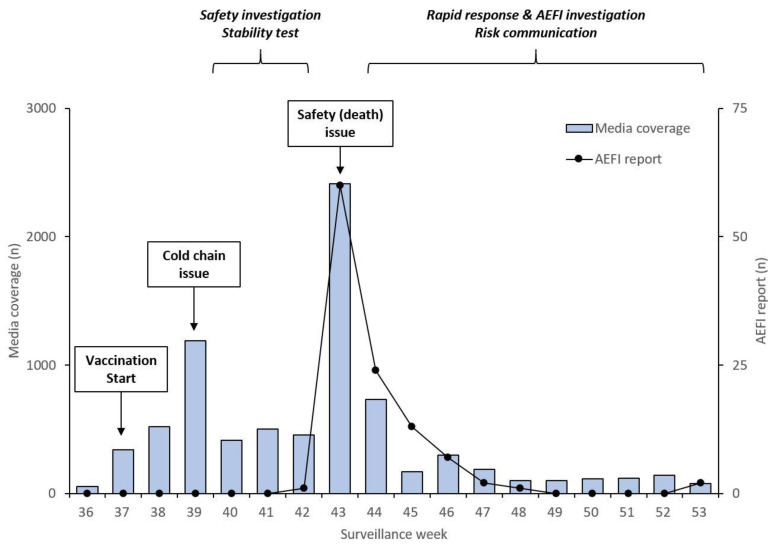
Weekly media coverage (**left axis**) and adverse events following immunization (AEFI) reporting (**right axis**) in South Korea in the 2020–2021 season.

Figure 2 shows the weekly media coverage of predefined keywords and AEFI reporting during the 36th and 53rd weeks of 2020. The national influenza vaccination campaign was started during the 37th week, while the cold chain issue was covered by the media during the 39th week of 2020. Thereafter, a safety investigation, including stability testing, was initiated. During the 43rd week of 2020, there was a large amount of media coverage on vaccine safety issues that framed the association between the influenza vaccine and the death of vaccinated individuals. A rapid response and AEFI investigation were initiated along with the risk communication issued by the government. The number of AEFI cases reported reached 60 in the corresponding week in which media coverage was in place during the 43rd week. The number of cases reported decreased to 24 and 13 in the following weeks.

Table 1 shows the mortality rate ratio for elderly individuals according to the vaccination status between 2015 and 2021. Between the 2015–2016 and 2019–2020 seasons, the mortality rate ratio for the influenza vaccines vs. non-vaccines ranged between 0.1296 (95% confidence interval (CI), 0.1262–0.1331, *p* < 0.0001) and 0.1608 (95% CI, 0.1572–0.1644, *p* < 0.0001). During the 2020–2021 season, the mortality rate ratio for influenza vaccines vs. non-vaccines was 0.1642 (95% CI, 0.1604–0.1681, *p* < 0.0001).

According to national statistics, the daily number of deaths between 2015 and 2019 was 597.7, and the average number of deaths between October and November 2019 was 646. When stratified according to the age groups who were eligible for vaccination, the numbers of deaths within 48 h were 432 in the vaccinated group and 722 in the unvaccinated group.

## 4. Discussion

In this study, we examined an abrupt increase in the AEFI reporting in the 2020–2021 season, likely associated with media coverage, which then caused a reporting bias in the passive surveillance system. Causality assessment between influenza vaccination and death was conducted for 108 reported cases during the 2020–2021 season, and none were found to have a plausible causal association with the vaccination [4]. Previous studies have examined the relationship between health rumors and news, affecting the vaccination coverage of essential vaccines. The vaccination coverage of the human papillomavirus (HPV) vaccine first declined in Japan after extensive news reports on AEFI after HPV vaccination [5]. The news then crossed borders with other countries and continents, causing global resistance to HPV vaccines [6]. Nonetheless, this incident was estimated to result in 5000 deaths from cervical cancer in Japan [7]. The current incidence in South Korea, in relation to the HPV vaccine safety issue, has caused an international impact that either alerted the national regulatory agencies or caused a temporary cessation of influenza vaccination [8,9].

We also found that the mortality rate was generally lower in those who were vaccinated with influenza vaccine throughout the surveillance seasons, which is inherent in the vaccine safety study due to a healthy vaccine effect [10]. The healthy vaccine effect phenomenon is characterized by lower relative mortality and morbidity rates due to selection bias, given that unhealthy individuals do not have an equal chance of receiving vaccines compared with healthy individuals, possibly masking an increased risk of death under study. Nevertheless, based on the longstanding science behind the vaccine’s effectiveness in reducing mortality associated with influenza, it is crucial to sustain a strong public vaccination program by responding to skepticism with epidemiological evidence. As in Italy, where the estimated chance of death within 48 h of vaccination was 15–20 individuals, the deaths following vaccination in Korea fell well within the daily expected number of deaths in the vaccinated elderly population [11]. Previous studies have shown conflicting results on whether influenza vaccination reduces all-cause mortality among the elderly population. During the 33 seasons from 1968 to 2001 in the United States, the all-cause excess mortality had no significant changes except during earlier years in A(H3N2)-dominated seasons [12]. Because few winter deaths are attributable to influenza in any season, the study concluded that observational studies demonstrated a vaccination benefit. Similarly, an Italian study from 1970 to 2001 showed no decline in age-adjusted excess mortality associated with increasing influenza vaccination distribution primarily targeted for the elderly population [13]. However, a meta-analysis of 20 cohort studies showed that influenza vaccination was 68% effective for preventing death and 27–30% effective for preventing deaths from all causes from three case-control studies, adding to the evidence that influenza vaccine reduces the risk of pneumonia, hospitalization, and death in elderly individuals during an influenza epidemic if the vaccine strain is identical or similar to the epidemic strain [14].

This study has several limitations. First, the assessment of the mortality rate ratio should be interpreted with caution, given the aforementioned healthy vaccine effect phenomenon [15]. Moreover, our study is subject to indication bias, in that the risk of death may be related to the indication for vaccine use, meaning that those who are under critical conditions not eligible for vaccination were not selected for vaccination against influenza [16]. Second, only the vaccination coverage and mortality data for a limited time span (early October to mid-November) were provided because this study was undertaken as a part of the rapid response to the vaccine safety crisis. Third, given that the follow-up time includes a period in which the vaccinated group cannot experience the outcome, the immortal time bias should be considered. Moreover, we found a significant difference in the mortality rate ratio between years with an upward trend since 2019. Given the relatively stable vaccination coverage between 2015 and 2019, there may be other unidentified factors affecting such differences, such as differences in circulating influenza virus strains and vaccine effectiveness in the respective seasons. Finally, given the unique situation of the coronavirus disease 2019 (COVID-19) pandemic during the 2020–2021 season, influenza vaccine procurement and distribution may have affected the initial roll out during the corresponding season. Despite this, our study provides a baseline mortality rate in the influenza vaccination target group of the elderly population in South Korea. We found an increased AEFI reporting rate following an increase in media coverage on vaccine safety. After hearing about the AEFIs in the media, there may have been hesitancy to receive the vaccine and refusal among the eligible population. An accurate information system in the media and a proactive risk communication channel may be warranted to foster increased trust in the public vaccination program.

The association between the influenza vaccine and death has been previously discussed elsewhere. In Italy, during the 2014–2015 season, the reporting of three deaths within 48 h of influenza vaccination [17], which concluded that there was a lack of causality between the reported deaths and the vaccination, caught the attention of the media and resulted in a 25–30% decrease in vaccine coverage [11]. As in Italy [18], our findings demonstrated the need for proper communication and collaboration between healthcare workers in the field of public health and health communication practitioners.

## 5. Conclusions

Vaccine safety surveillance should be continued in conjunction with thorough investigation and transparent risk communication, which will help maintain public trust in vaccines and vaccinations. Experiences of adverse events caused by influenza vaccination in South Korea may aid in the development of preparedness and response measures during mass vaccination programs for COVID-19.

## Figures and Tables

**Table 1:** Mortality rate ratio for elderly individuals aged ≥65 years by vaccination status in South Korea between 2015 and 2021.

Seasons	Vaccinated		Unvaccinated		MRR **	(95% CI)	*P*
	Mortality Rate *	(95% CI)	Mortality Rate *	(95% CI)			
2015–2016	157	(154–161)	1213	(1194–1232)	0.1296	(0.1262–0.1331)	<0.0001
2016–2017	193	(189–196)	1280	(1261–1301)	0.1504	(0.1468–0.1541)	<0.0001
2017–2018	160	(156–163)	1360	(1341–1380)	0.1174	(0.1145–0.1203)	<0.0001
2018–2019	177	(174–180)	1313	(1294–1333)	0.1346	(0.1314–0.1378)	<0.0001
2019–2020	208	(205–212)	1184	(1276–1315)	0.1757	(0.1572–0.1644)	<0.0001
2020–2021	166	(163–169)	1009	(996–1023)	0.1642	(0.1604–0.1681)	<0.0001

* Per 100,000; from the first week of October (usual start of the influenza vaccination campaign for the corresponding season) to the second week of November. ** MRR, mortality rate ratio.

## Data Availability

The data used in this study is protected under the Personal Information Protection Act.
